# Seasonal patterns in the mesopelagic fish community and associated deep scattering layers of an enclosed deep basin

**DOI:** 10.1038/s41598-023-44765-5

**Published:** 2023-10-19

**Authors:** Z. Kapelonis, A. Siapatis, A. Machias, S. Somarakis, K. Markakis, M. Giannoulaki, N. Badouvas, K. Tsagarakis

**Affiliations:** 1 Institute of Marine Biological Resources and Inland Waters, Hellenic Centre for Marine Research (HCMR), Athens, Greece; 2 Institute of Marine Biological Resources and Inland Waters, Hellenic Centre for Marine Research (HCMR), Heraklion, Crete Greece

**Keywords:** Community ecology, Ecosystem ecology, Ichthyology, Marine biology

## Abstract

Mesopelagic fish constitute the most abundant vertebrate group in the marine environment. The current work reports on results of three seasonal acoustic cruises carried out in the Gulf of Corinth, a relatively small, deep, isolated basin located in the Central Mediterranean (Greece) that presents some unique geomorphological and ecological features. The aim of this study was to describe seasonal echo-types and the vertical distribution of the Deep Scattering Layers (DSLs) as well as to relate them with specific species or species groups. Mesopelagic fish dominated the pelagic ecosystem as confirmed by biological sampling with different gears during daytime and nighttime. In total, at least 15 species were caught, belonging to the families Myctophidae, Paralepididae, Sternoptychidae and Stomiidae, while the—elsewhere very abundant—families Gonostomatidae and Phosichthyidae were completely absent. Common echo-types included: (a) shoals and schools formed by the silvery lightfish *Maurolicus muelleri*, usually located along the shelf break (80–225 m)*,* (b) a non-migrant thin DSL found at 150–280 m throughout the deep parts of the Gulf, dominated by juvenile half-naked hatchetfish *Argyropelecus hemigymnus*, and (c) one thick, partially migratory DSL at 250–600 m, mainly consisting of myctophids. The echo backscatter characteristics and species composition of the DSLs as well as the length distribution of the populations were found to differ seasonally. Species-specific and size related patterns in the vertical distribution of fish were detected both during daytime and nighttime. Overall, the Gulf of Corinth seems to sustain high densities of mesopelagic fish that constitute the basic food resource for the abundant dolphin populations that inhabit the area.

## Introduction

Mesopelagic fish inhabit almost all seas where depths exceed 200 m (sometimes even shallower^1^), and may be distributed down to 1000 m in the water column, forming Deep Scattering Layers (DSLs) detected by echosounders^1,2^. In this bathymetric range, i.e. the twilight zone, light is insufficient for photosynthesis, but still not completely absent and is sufficient for vision^1^. The mesopelagic fish fauna and the organisms of the mesopelagic zone in general have attracted much attention recently^3^, mainly because it is now documented that their global abundance had been previously underestimated^4,5^. They participate in a large share of the biogeochemical fluxes in the oceans^6^ as they are major mesozooplankton consumers and constitute important prey for sub-apex and apex predators^7^. Several species perform Diel Vertical Migrations (DVM) moving to shallow water layers during the night to feed, and returning to the mesopelagic zone at dawn. Through this behaviour they function as a link between the surface and deeper layers and constitute an important component of the biological pump, i.e. the mechanism of carbon sequestration by the oceans^6^.

Although the open ocean is often perceived as an environment with few barriers, the composition and abundance of the mesopelagic fish communities varies greatly according to latitude^8^, climatic conditions, local productivity^9^ and oceanographic features^10^, and so may be true for the populations’ condition^11^ and other biological parameters. The composition of the mesopelagic fish communities may also differ vertically^1,12^, shaped by bathymetric preferences and behavioural traits (e.g. DVM) of the constituting species. It is therefore not surprising that bathymetry and topography, along with the features mentioned above, affect the presence of species and their relative abundance^13^. In addition to these, seasonal patterns related to abiotic as well as life histories and ontogenetic changes have been identified in arctic^14^ and temperate^15,16^ regions.

Despite the challenges and high uncertainties in their application in the mesopelagic zone^17^, acoustics are the main tool for studying mesopelagic fish behaviour, horizontal and vertical distribution of the DSLs and for inferring biomass estimates. Biological sampling is considered necessary to reduce this uncertainty; however, this is also accompanied by several issues, such as efficient trawl avoidance^18^ as well as differing catchability among species and sampling gears. Routine surveys targeting mesopelagic fish are generally lacking because of their low commercial interest, resulting in limited common practices, coordination and intercomparison among surveys^19^.

In the Mediterranean Sea, although there are some works providing information on the biology of mesopelagic fishes^e.g.^^20–22^, targeted studies on the mesopelagic fish communities are limited and mainly refer to the western basin^15,23–25^. In the eastern basin, to our knowledge, the only relevant studies concern ichthyoplankton surveys which provided insights on the species composition in several Greek seas and gulfs^26^ and a preliminary survey targeting plankton and (mainly) juvenile fish assemblages of the North Aegean Sea mesopelagic zone^27^. In the current work, we present the results of three seasonal acoustic surveys conducted in an enclosed deep basin, the Gulf of Corinth (Central Mediterranean, Greece), with the aim to (i) identify different echo-types and analyze their bathymetric distribution, (ii) explore their species composition with targeted hauling using various gears, (iii) describe aspects of DVM occurring in the study area, and (iv) study seasonal specificities.

## Materials and methods

### Study area

**Figure 1:**
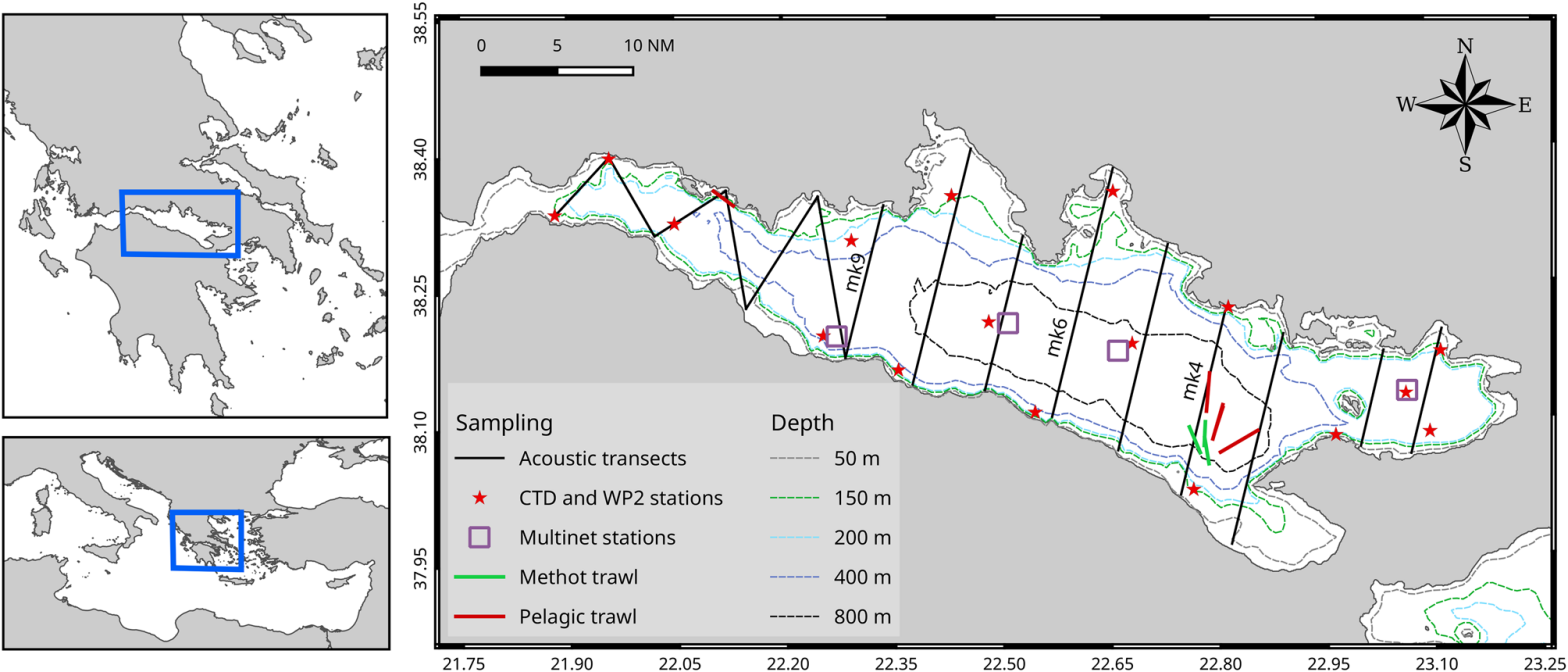
The Gulf of Corinth, bathymetry and sampling design. Acoustic transects were the same in all cruises, while Multinet and CTD stations as well as haul locations refer to the first survey (November 2018). Text labels (mk4, mk6, mk9) encode specific transects referenced in other figures. Maps were produced with software QGIS 3.28 (https://qgis.org).

The Gulf of Corinth (Greece; Fig. 1), located at the east end of the Ionian Sea, is characterized by a narrow continental shelf and a steep continental slope ending in a deep plateau with a maximum depth of 935 m. It is an enclosed, 130 km long, semi-isolated basin, connected to its west with the Ionian Sea through the relatively shallow Patraikos Gulf (maximum depth ~ 140 m), while an even shallower strait (< 50 m) connects the two Gulfs. The hydrography of the Gulf of Corinth has not been extensively studied; deep-water masses of the Gulf are characterized by lower temperature and salinity compared to the open Ionian Sea because they are formed locally in the Gulf and there is limited exchange with the open sea^28^. No oxygen minimum zones are reported in the region^26,28^. To the east, the Gulf of Corinth communicates with the Aegean Sea through the Corinth Canal, an artificial 6.3 km long and shallow (8 m depth) canal, constructed in the late nineteenth century. The region presents a special interest from a geological^29^ as well as an ecological perspective. It is listed as a Site of Community Importance in the NATURA 2000 Network and an IUCN Important Marine Mammal Area^30^ as it hosts several important habitats and species of conservation concern. Among these, the most emblematic are the dolphins that the Gulf of Corinth sustains to relatively large abundance^30,31^. Specifically, common bottlenose dolphins, *Tursiops truncatus*, striped dolphins, *Stenella coeruleoalba*, and common dolphins, *Delphinus delphis*, are permanent residents of the area, while extensive interactions between the latter two are observed, including the formation of mixed schools and probable hybridization^30^. Especially the common dolphin subpopulation of the Gulf of Corinth is considered critically endangered^32^, which raises the need for further focused research and immediate management actions. The pelagic environment of the Gulf has also faced jellyfish blooms, mainly of the mauve stinger, *Pelagia noctiluca*, with adverse effects on touristic and fishing activities in the area. Whether such blooms are periodic or occasional remains uncertain, and so it remains to be examined if and how they are influenced by environmental drivers and/or interspecific interactions, namely competition and predation. Within the pelagic environment of the Gulf, the role of the mesopelagic fish community is unclear but possibly important due to the extent of deep regions and the low abundance of other forage fish in the Gulf^33^. Previous ichthyoplankton studies have reported the existence of a significant number of mesopelagic fish species^26^, despite its geographical isolation from other deep basins; however the mesopelagic fish adult community has not been studied.

### Acoustic data

Three acoustic surveys took place on-board the R/V PHILIA in the Gulf of Corinth (Fig. 1), in November 2018, April 2019 and October 2019, in order to study the DSLs and the mesopelagic fish species associated with them. Acoustic data were collected with a speed of 8 kn using a hull-mounted SIMRAD EK80 echosounder operating four split-beam transducers at 38, 120, 200 and 333 kHz. Only the 38 kHz frequency is operational at all surveyed depths (maximum depth is 935 m) and was the only one used in the analysis. The transducer (SIMRAD ES38-7) was operated in continuous-wave (CW) mode with fast ramping and a pulse duration of 1.024 ms, the ping rate was varying and set at the maximum value allowed by the local bottom depth. The system was calibrated for the chosen acquisition settings using the standard target method^34^ prior to each survey. Acoustic sampling was performed along nine predefined parallel transects 5 nmi apart in the middle of the Gulf, while due to topography peculiarities in the western and narrower part of the Gulf six additional zig-zag transects were held (Fig. 1). Acoustic data on the transects were acquired during daytime, while additionally, echo was recorded during night to capture the fish DVM.

### Biological sampling and oceanographic data

In order to obtain biological samples the following hauling equipment was used (Table 1): (i) a pelagic trawl (hereafter “Trawl”) with vertical opening 7 m, horizontal opening 12 m and a 16 mm (stretched) mesh cod-end, typically towed at 3–4 knots, (ii) a SARDONET pelagic trawl (hereafter “Sardonet”) with vertical and horizontal opening 2.1 m and 3.7 m respectively and 5 mm size mesh at the cod-end, also towed at 3–4 knots, (iii) a Methot frame trawl^35^ (typically towed at 2 knots) with a 1.5 m side and 1 mm mesh net leading to a large bottle with a fine mesh and (iv) a Multinet plankton sampler (square with a 0.5 m side, typically towed at 3 knots) with five 300 μm nets leading to bottles with the same mesh. Deployment of towed gears was monitored using a SCANMAR depth sensor, while in the pelagic trawl a TrawlEye sensor was also attached to the head-rope to monitor the opening.

**Table 1:** Specifications of the hauling equipment used for biological sampling.

	Trawl	Sardonet	Methot frame	Multinet
Horizontal opening (m)	12	3.7	1.5	0.5
Vertical opening (m)	7	2.1	1.5	0.5
Stretched mesh size	16 mm	5 mm	1 mm	300 μm
speed (knots)	3–4	3–4	2	3
usage	Main sampling gear for fish	Backup gear and exploratory deployment	Mainly for juveniles, small sized species and ichthyoplankton	5 nets, plankton sampling in different layers

The Sardonet trawl was used only in two hauls as a back-up gear due to failure of the regularly used pelagic trawl in April 2019, but at the same time this allowed a comparison of the catch efficiency among gears. After deployment of all gears in the first cruise, it was decided that the pelagic trawl was the go-to gear for echotrace identification; its small opening (compared to common pelagic trawl gear) allows for accurate sampling of even small acoustic structures, such as thin layers. The other two sampling gears were used to characterize the ecosystem at smaller scales ranging from plankton to larval, juvenile fish and small-sized fishes (Table 1). At the same time, catch information from these gears was useful to identify if scatterers other than fish contributed to the mesopelagic acoustic layers, or if they produced their own echo-types.

The sampling locations (Fig. 1) and depths were decided ad-hoc, based on the observed echotraces. The number of hauls performed with each gear as well as the number of CTD stations are shown in Table 2. The first survey (November 2018) was also exploratory, hence all gears (with the exception of Sardonet trawl as mentioned above) were hauled to targeted echo-types to facilitate the decision on gear suitability, based on the catch composition and quantity. In the following surveys, the use of sampling gears was more selectively applied based on progressively gained experience and time constraints. Hauls were performed in daytime in order to sample the most evident scattering layers, as well as during the night to explore species composition in the migratory (i.e., near surface hauls) and the non-migratory (i.e., deeper hauls) layers. In all surveys, at least one trawl (or Sardonet) haul was performed in the upper (< 300 m) and lower (> 300 m) layers during daytime as well as in the upper (< 100 m) and lower (> 250 m) layers during nighttime. The only exception was in October 2019 that the lower layers (> 250 m) were not sampled during nighttime. Haul duration ranged between 40 and 70 min.

**Table 2:** Number of hauls performed with each sampling gear and CTD stations in each sampling period.

	November 2018	April 2019	October 2019
Pelagic trawl	5	6	4
Sardonet trawl	–	2	–
Methot frame	4	1	1
Multinet hauls	4	4	-
CTD stations	17	13	19

Trawl catches were sorted on-board to species level, when possible, total weight per species was measured and total numbers were counted or estimated based on a sub-sample and total species’ weight. Total length (TL) per individual was also measured for up to 100 specimens in order to obtain an estimate of the length frequency distribution per haul. When species identification was uncertain, samples were transferred to the lab for further identification. Samples from Methot and Multinet hauls were preserved in formalin and transferred to lab for sorting. The analysis focused on fish, as other organisms (e.g. cephalopods) were not regularly present in hauls, potentially due to low gear catchability.

A Sea-Bird SBE 19plus V2 SeaCAT CTD Profiler was used to collect hydrographic parameters at different locations of the surveyed areas (13 stations in November 2018, 12 stations in April 2019 and 14 stations in the October 2019 cruise). Parameters collected included conductivity, temperature, dissolved oxygen and fluorescence. The CTD profiler was deployed at each station from 1 m below surface to 5 m above the seafloor, or to a maximum depth of 150 m if the station was situated at a deeper location. As an exception, in each survey, a single station situated at a location with depth close to the maximum gulf depth (approximately 850 m), was held close to the seafloor.

The law pertaining to the operation of ethics committees within research centers in Greece was prescribed in 2018 (Ν4521/2018, Government Gazette Issue 38/Α/2018) and adapted by the HCMR administrative council decision (415/Γ12-18.11.2022), both enacted after the outset of the program which implemented the survey design for this work; therefore, there was no obligation or provision for the approval of the sampling plan. Still, all biological sampling was performed in accordance with standard guidelines for experimental procedures in field surveys^36^, also following the protocol used in the MEDiteranean International Acoustic Survey (MEDIAS), a survey targeting small pelagic fish^37^. Methods are reported in accordance with the ARRIVE guidelines^38^ which are relevant for field research with wild animals.

### Data analysis

The species composition per haul was estimated (and presented as percentage by number) aiming to identify the main species constituting each DSL/echo-type and derive conclusion on the diel bathymetric distribution of the species.

In addition, fish length data were analyzed to study the catch efficiency of the gears as well as bathymetric, diel and seasonal patterns in the populations. The species’ length frequency distribution (LFD) was estimated per (i) echo-type and depth stratum (Upper/Lower) during Day and Night, (ii) cruise and (iii) sampling gear (i.e. Trawl/Sardonet/Methot). For these estimates, the length frequencies of the constituting hauls were pooled after weighting with the number of individuals caught per hour of hauling. The LFD of the most abundant species caught, were presented as density plots and inspected to compare the size-related catch efficiency of the three sampling gears. The length distribution statistics of the species in the different layers were presented as box-plots to identify size-related differences in bathymetric distribution and DVM behaviour for each species. LFD density plots per species in each research cruise were also produced in order to identify seasonal patterns related to species’ life histories and explore whether the LFDs of the autumn cruises (November 2018 and October 2019) were more similar to each other compared to the one held in spring (April 2019).

Echogram scrutinization took place in two stages, preprocessing and main analysis. In the preprocessing stage, the raw acoustic data were grouped per-transect and the calibration was tuned for the local environment based on CTD cast information (temperature and salinity). The sea bottom line was defined using an automatic bottom detection algorithm with a backstep of 0.5 m to account for the bottom dead-zone, and manually corrected where needed. A surface exclusion zone of 7 m from the transducer face (approximately 10 m from sea surface) was defined to eliminate data with acoustic near-field effects. Regions not appropriate for analysis due to noise or other artefacts were manually excluded from further analysis. Background acoustic noise was removed by means of the algorithm^39^ implemented in Echoview^®^ software^40^ version 10.0.320, using a horizontal extent of 25 pings, vertical extent 10 m, vertical overlap 25%, maximum noise − 125 dB and signal-to-noise ratio (SNR) threshold 10 dB. A threshold value of − 80 dB for the mean volume backscattering strength (S_v_) [dB re 1 m^−1^] was used throughout the analysis.

In the main analysis stage, echograms for all transects were collectively reviewed and the dominant echo-types (including different DSLs) were identified. Each echotrace was defined by means of a manually outlined polygon, and assigned to the appropriate echo-type. For the statistical quantification of the vertical distribution and backscatter of each echo-type, and Elementary Distance Sampling Unit (EDSU) of 300 m was selected after testing levels from 25 up to 1000 m in order to conclude to the smaller EDSU beyond which no substantial difference in the estimated indices occurred. For each interval defined by the EDSU and each one of the defined regions, a number of descriptors were calculated based on the depth (d) [m] and the linear-domain volume back-scattering coefficient (s_v_) [m^2^ m^−3^] of the acoustic samples composing the region (within the specific interval):

i. Mean depth [m]: the mean value of d, weighted by the values of s_v_.
ii. Thickness [m]: the range between the 0.5% and 99.5% percentiles of the distribution of d, weighted by the s_v_.
iii. Altitude index [unitless]: ratio between the Mean depth of the DSL and the average seafloor depth for the specified interval.
iv. Nautical Area Scattering Coefficient (NASC-s_A_) [m^2^ nmi^−2^]: The acoustic backscatter (s_v_) integration of the region samples for the specified interval scaled to 1 nmi^2^.

In order to quantify the DVM effect on the acoustic backscatter, acoustic recordings collected during the biological sampling of the November 2018 cruise were stratified in three depth zones (0–150, 150–300, 300–800 m) and each zone integrated (EDSU 300 m) to calculate its average NASC and standard deviation during day and night. A minimum distance of two hours from sunrise/sunset was used to separate day and night data.

Finally, the vertical profiles of the environmental parameters (temperature, salinity, oxygen concentration, fluorescence) in each cruise were calculated by averaging the values in all CTD stations. The profiles were used to explore community patterns in each cruise in relation to seasonal environmental variations.

Echogram preprocessing and region classification took place in Echoview^®^ software^40^ version 10.0.320, while Python (with NumPy, SciPy and Matplotlib libraries)^41–43^ was used for the statistical analysis of the layer data and the CTD profiles. For the analysis of biological sample data, the R language^44^ was used. Μaps were produced with QGIS software^45^ version 3.28 using bathymetric and coastline data extracted from the GEBCO_08 grid^46^.

## Results

**Figure 2:**
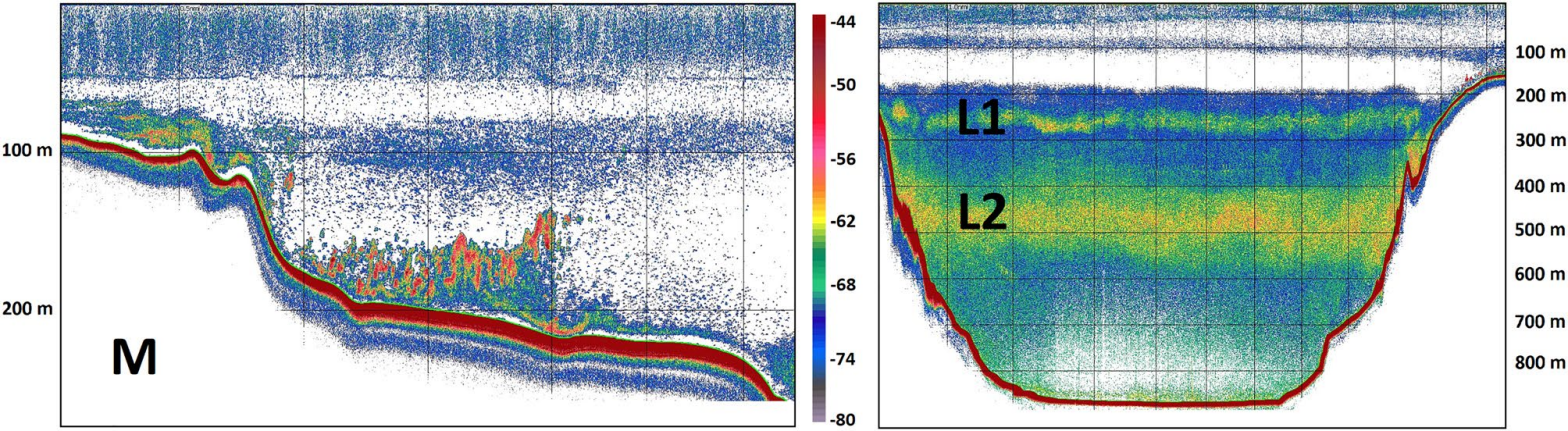
Echograms recorded during the November 2018 sampling cruise, exemplifying the main echo-types (used also for the acoustic data analysis of the other two surveys). M echogram from North section of transect mk9 (North–South direction, 15 Nov. 2018, 8:54–9:29 UTC), L1/L2 echogram from the entire deep section of mk6 (South-North direction, 14 Nov. 2018, 7:52—9:28 UTC) (see Fig. 1), grid lines arranged by 100 m in depth and 1 nmi along transect intervals for both panels. Echogram images extracted with Echoview^®^ 10.0.320.

Mesopelagic fish dominated the pelagic ecosystem of the Gulf, as evidenced by the distribution of backscatter which extended in several depth strata all along the basin (Fig. 2, Suppl. Fig. S1). The dominance of mesopelagic fish was confirmed by sampling the scattering layers and echotraces of the mesopelagic zone with the three trawls (Trawl, Sardonet and Methot) while the qualitative assessment of Multinet samples at the same layers could not justify the measured backscatter. In total, at least 15 fish species were caught (Trawl: Table 3; Methot frame: Suppl. material Table S1), belonging to the families Myctophidae, Paralepididae, Sternoptychidae and Stomiidae, while the—elsewhere very abundant^25^—families Gonostomatidae and Phosichthyidae were completely absent.

**Table 3:** Haul characteristics and catch composition (numbers h^−1^) in each haul.

Cruise	Nov 2018					Apr 2019								Oct 2019			
D/N	D			N		D						N		D			N
Depth (min; m)	100	226	464	19	507	122	167	218	321	46	669	230	40	124	206	533	29
Depth (max; m)	225	243	545	30	570	139	187	251	330	68	725	249	56	154	207	570	70
Stratum	Upper	Upper	Lower	Upper	Lower	Upper	Upper	Upper	Lower	Upper	Lower	Lower	Upper	Upper	Upper	Lower	Upper
Species|Station	TR17	TR3	TR4	TR1	TR2	TR2	TR1	TR5	TR4	TR3	TR6	SAR2	SAR1	TR4	TR1	TR2	TR3
Myctophidae																	
* Benthosema glaciale*		2.9	118.4	456.7	550.5			27.7	65.9		**781.4**	**124.4**	**395.7**			**692.2**	
* Ceratoscopelus maderensis*		38.7	**172.1**	**9833.9**	**2418.9**			2.3	77.6		**289.1**	61.5	**294.9**			**263.5**	**688.9**
* Diaphus holti*		12.6	22.1		91.6		4.8	**177.7**	30.6		53.2	11.7			**87.4**	21.7	
* Diaphus rafinesquii*			1.6														
* Hygophum benoiti*			129.5		152.6			6.9	**242.4**		72.3	7.3	46.0			16.5	118.9
* Lampanyctus crocodilus*		1.0	41.1		10.5				23.5		182.7	2.9				183.5	
* Notoscopelus *sp.			178.4	84.5	214.7			5.8	7.1		13.6		23.0			251.3	5.6
* Myctophum punctatum*			**383.7**					1.2	52.9		13.6		35.7			183.5	65.6
Sternoptychidae																	
* Maurolicus muelleri*	**7314.0**					**6076.9**	**9039.6**	135.0	23.5	**22.0**	1.4			**5500.0**	7.4		
* Argyropelecus hemigymnus*		**87.1**	45.8		102.1			**379.6**	**121.2**		162.3	**144.9**	1.3		31.6	63.5	15.6
Paralepididae																	
Paralepididae		8.7															
* Lestidiops *sp.				9.8	6.3		1.2	47.3	62.4		140.5						1.1
* Lestidiops jayakari*		2.9	4.7		1.1					4.9							
* Paralepis coregonoides*					1.1												
* Paralepis speciosa*					10.5												
* Arctozenus risso*							1.2	4.6	1.2		5.5					0.9	
Stomiidae																	
* Stomias boa*		5.8	9.5		9.5				2.4		10.9	2.9	6.4			9.6	2.2
Total	7314.0	159.6	1106.9	10,384.9	3569.4	6076.9	9046.8	788.1	710.6	27.0	1726.4	355.5	802.9	5500	157.9	1690.4	897.8

The species cumulatively contributing > 50% in each haul are marked in bold. Stations SAR1 and SAR2 were sampled with Sardonet trawl. D: day; N: night.

**Figure 3:**
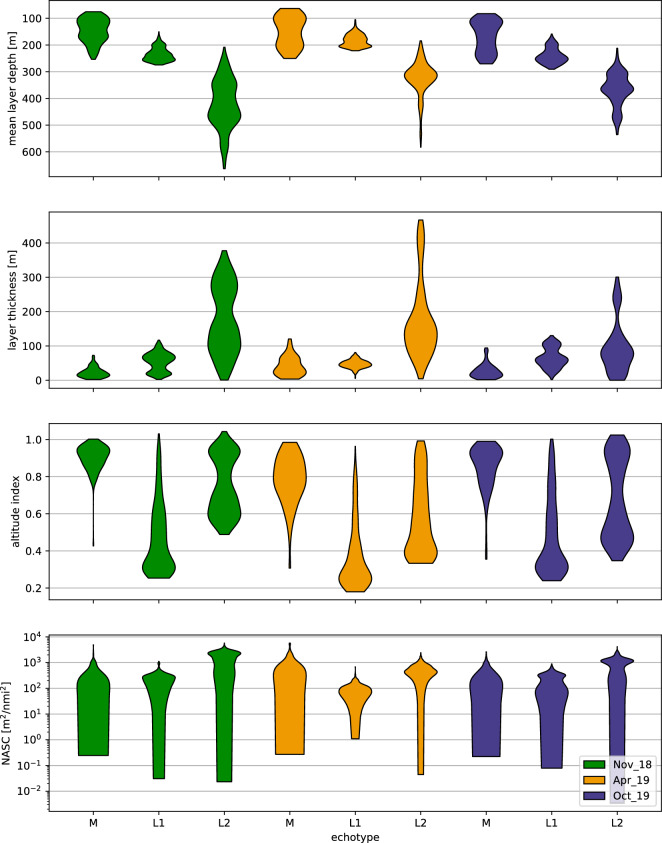
Violin plots of the characteristics of the main echo-types identified in each season during daytime: mean depth; thickness; altitude index; Nautical Area Scattering Coefficient (NASC). EDSU is 300 m. Horizontal axis indicates different echo-types mentioned in the text and shown in Fig. 2.

One of the main echo-types detected (hereafter named “M”) included schools usually located a few meters above the seabed along the shelf break, while shoals in close association to these schools were also present in shallower adjacent areas (Figs. 2, 3). The schools and shoals were formed by the silvery lightfish *Maurolicus muelleri*, as revealed by the catch composition of the hauls targeting them (TR17 in November 2018, TR1 and TR2 in April 2019 and TR4 in October 2019; Table 3). In addition, there was a clear size-related pattern in the depth distribution of *M. muelleri* with smaller and larger individuals located at shallower and deeper waters respectively (Suppl. Figs. S1 and S2) during the day. The M echo-type exhibited similar characteristics in all three cruises, distributed between 80 and 225 m (mean depth 140 m) with a thickness of 28 m (Fig. 3).

In addition, other typical DSLs were present in the basin, vertically located between approximately 200 and 800 m depth and horizontally distributed all over the Gulf, where allowed by the local bathymetry (Figs. 2, 3). Depending on the season, 2–3 typical DSLs were observed, but for the sake of the analysis, their number was reduced to two, after taking into account the vertical distribution of the biological sampling. Contrary to echo-type M, the DSLs were formed by several species, while their composition might change seasonally. A shallower DSL (hereafter L1) was present at ~ 250 m in autumn samplings (November 2018, October 2019) and at ~ 180 m during the spring cruise (April 2019), while its thickness was narrower in spring (52 m) than in autumn (62–78 m; Fig. 3). L1 was composed of mainly juvenile half-naked hatchetfish *Argyropelecus hemigymnus* and few more species that varied seasonally (mainly small lantern fish *Diaphus holti* in April and October 2019 and Madeira lantern fish *Ceratoscopelus maderensis* in November 2018; Table 2).

The deeper DSL (hereafter L2) was inhabited by a mix of mesopelagic species, mainly myctophids. In November 2018 and October 2019, L2 was found at similar depths (455 m) with a thickness of 110 m and 200 m respectively. In April 2019, the corresponding layer had a mean depth of 320 m and thickness 160 m.

In addition to the above, a shallow (~ 50 m) strongly backscattering layer was present all along the Gulf in April 2019 but was not taken into consideration as it included post-larvae and small juveniles *M. muelleri*, along with other species’ juveniles and larvae, as confirmed by a Methot frame tow (Suppl. material Table S1).

**Figure 4:**
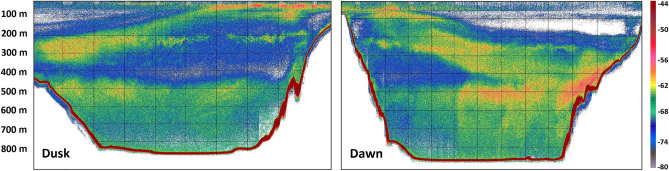
Echograms along transect mk4 (see Fig. 1) recorded during the November 2018 sampling cruise showing DVM of mesopelagic fish DSLs during dusk (North–South direction, 10 Nov. 2018, 15:27–16:40 UTC) and dawn (South-North direction, 11 Nov. 2018, 6:19–7:59 UTC), grid lines arranged by 100 m in depth and 1 nmi along transect intervals. Echogram images extracted with Echoview^®^ 10.0.3201.

**Figure 5:**
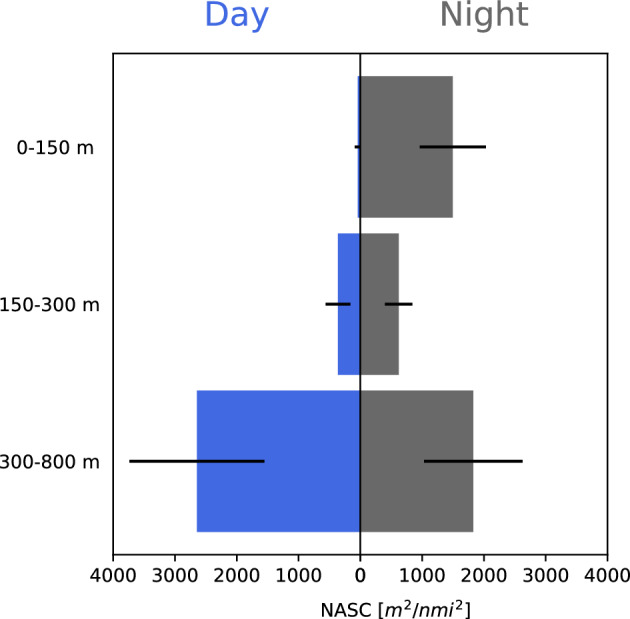
Day vs night average NASC and st. dev. for three depth strata: 0–150, 150–300, and 300–800 m, based on echograms recorded during the biological sampling of the November 2018 cruise and an EDSU of 300 m.

The spatial allocation (mapped as NASC per 1 nmi EDSU) of the abovementioned echo-types in the Gulf during November 2018 is shown in Suppl. Fig. S2. It is apparent that *M. muelleri* shoals and schools (M echo-type) were restricted near the coast, over the shelf break. Contrary, the L1 and L2 were distributed in the centre of the Gulf, in all areas where the depth was deep enough to support their presence (Suppl. Fig. S1). Dusk and dawn acoustic recordings in the area (Fig. 4) were dominated by the partial migration of L2 which reached near the surface, while L1 was mostly non-migratory. The pattern is also supported when observing the nocturnal shift in the depth-stratified average NASC values (Fig. 5). The total backscatter in the deeper zone (corresponding to the depth range of echo-type L2) is reduced by 30% during the night with a simultaneous substantial increase in the surface zone (where mesopelagic species were caught only during the night), while the intermediate zone 150–300 m (depth range of echo-type L1) also displays an increase in backscatter, likely due to the crossing migrators from deeper layers.

**Figure 6:**
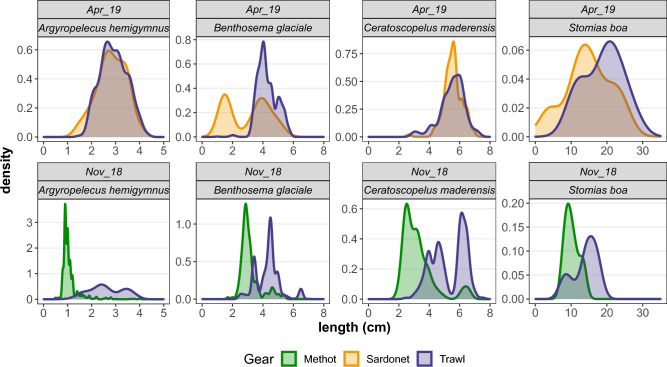
Probability densities of length frequency distribution for the most abundant species caught with Trawl, Sardonet and Methot frame.

The LFD of the species caught with the different sampling gears (Fig. 6) revealed that the catch of the Methot frame was dominated by smaller individuals, mostly larvae and, to a lesser extent, juveniles (Suppl. Material Table S1). The midwater Trawl and the Sardonet trawl caught similar sizes, depending also on the species (Fig. 6); for example, the midwater trawl caught larger individuals of *Benthosema glaciale* and *Stomias boa* while for *A. hemigymnus* and *C. maderensis* the length range caught was similar.

**Figure 7:**
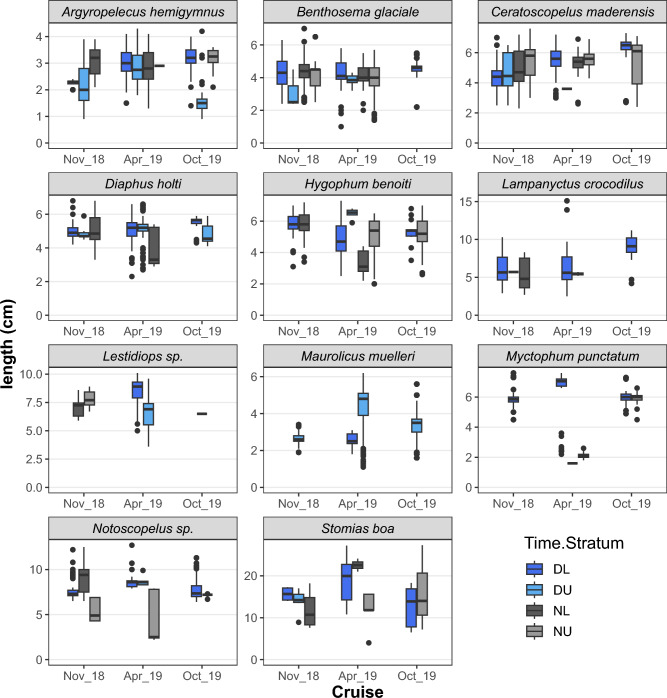
Box-plots for fish lengths (TL) recorded in each stratum during day and night. DL: daytime in lower layer; DU: daytime in upper layer; NL: nighttime in lower layer; NU: nighttime in upper layer. The DU layer was considered to include echo-types located at depths shallower than 300 m (i.e. M and L1), while DL layer included deeper echo-types (i.e., L2). During nighttime, NU layer (< 100 m) was considered to include migratory populations, while NL layer included the non-migratory populations staying at deeper layers during the night.

**Figure 8:**
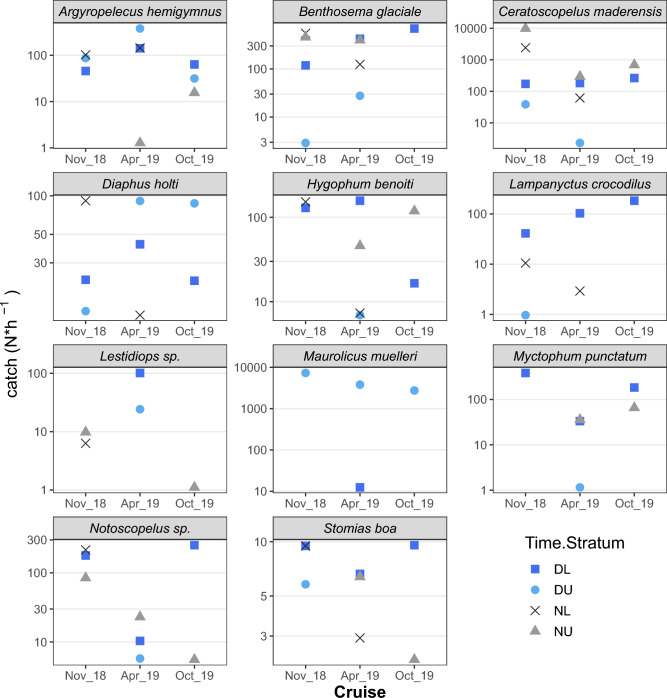
Mean number of individuals (log scale) caught per hour of trawling in the different depth strata during day and night. DL: daytime in lower layer; DU: daytime in upper layer; NL: nighttime in lower layer; NU: nighttime in upper layer; see Fig. 7 for layer/echo-type association.

The length distribution statistics of the species and the mean numbers caught in the different layers during day and night are presented in Figs. 7 and 8 respectively. Collectively, these figures can be used to identify size-related differences in depth preferences and DVM behaviour for each species. During the Day, the Upper layer (i.e. DU) was considered to include M and L1 echo-types which were located at depths shallower than 300 m, while the Lower layer (DL) included L2 echo-type. During Nighttime, the Upper layer (< 100 m; NU) was considered to include migratory populations while the Lower layers (NL) included the non-migratory populations. Smaller individuals of *A. hemigymnus* inhabited DSLs located in shallower waters during the day and a similar pattern was observed for *D. holti*, especially for the October 2019 cruise (Fig. 7)*.* These two species, which didn’t seem to migrate to upper layers during the night (Fig. 8), were the main ones forming L1, i.e. the shallower (~ 200 m) daytime DSLs; however, at the same time, larger individuals of both species were also present in deeper layers, as shown by the catch composition of the hauls (Table 2, Figs. 7 and 8). Similarly, for *B. glaciale*, larger individuals were observed in deeper waters both during daytime and during nighttime. During the night, larger individuals of *C. maderensis, H. benoiti* and *Lestidiops *sp. were recorded in shallower waters while the contrary was observed for *Notoscopelus *sp. (Fig. 7). *Myctophum punctatum* was mainly caught during the day in lower DSLs but during the night it was absent from deep layers and only few individuals were caught in shallow waters (Fig. 8), probably because it migrates up to the surface, where no trawling took place. The only other species not caught in the upper layers during the night was *Lampanyctus crocodilus.* It should be noted that *M. muelleri* is absent from the night hauls because no night sampling was performed at sites were echo-type M was detected during the day.

**Figure 9:**
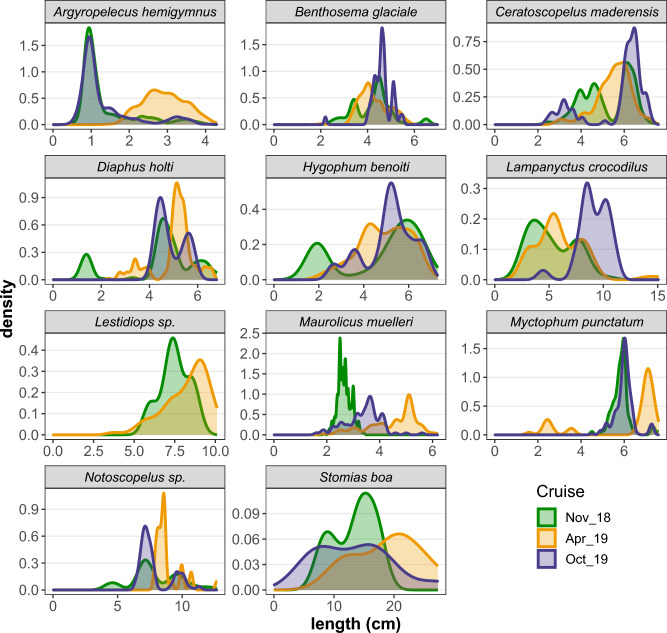
Probability densities for fish lengths (TL) in each cruise for species caught in adequate numbers.

Seasonal LFDs clearly showed that for almost all species, the population structure was very similar between November 2018 and October 2019, and quite different compared to April 2019 (Fig. 9). The few exceptions concerned *L. crocodilus* and *M. muelleri;* however, for the latter, the divergence between the two autumn surveys had to do with the fact that samplings didn’t cover all the depth range of the species, and therefore not all sizes were adequately sampled. For several species (e.g. *D. holti*), a bimodal or multi-modal LFD was apparent in one or both seasons, implying the presence of different age groups.

**Figure 10:**
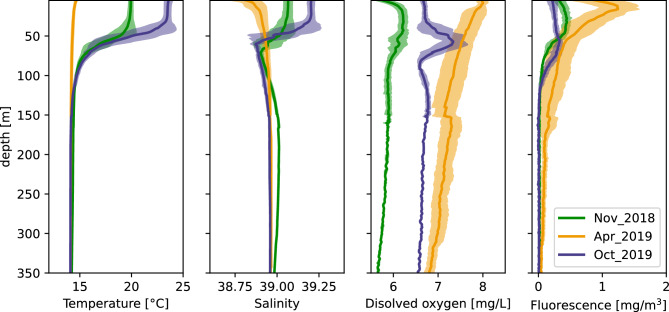
Vertical profiles of temperature, salinity, oxygen concentration and fluorescence in the three sampling cruises. Lines show the mean profile for all stations of each survey and the area plot corresponds to ± one standard deviation. Only depths up to 350 m are shown for better illustrative reasons and because all environmental parameters were very stable below this depth. All casts were performed during daytime concurrently to the acoustic sampling.

The inspection of the vertical profiles of temperature, salinity, oxygen concentration and fluorescence showed that environmental conditions in November 2018 were very close to the ones in October 2019 (Fig. 10). In addition, a clear surface stratification pattern was observed in autumn (both cruises), contrary to spring. Fluorescent peaked in surface waters in April while maximum values were found at ~ 60 m in autumn surveys. The dissolved oxygen concentrations were quite high in all seasons and did not seem to restrict the vertical distribution of mesopelagic fish. Below 100 m depth, all parameters were quite stable.

## Discussion

Research on mesopelagic fish is at relatively early stage with a growing interest in recent years due to their ecological importance and potential use as a fisheries resource^47^. The current work presents the results of the first acoustic survey aiming to study DSLs and their composition, not only in the Gulf of Corinth, but also in the Central Mediterranean Sea. The fishes diversity of the mesopelagic zone in the study area is relatively low, as has been previously reported for the Mediterranean^10,15,25^ where the presence of many deep-water species inhabiting the adjacent eastern Atlantic seems to be constrained by (i) the relatively shallow strait (Gibraltar) connecting the two Seas and (ii) the high temperature in the deep waters of the Mediterranean compared to the colder Atlantic^23,48^. In addition, no Mediterranean members of the Gonostomatidae and Phosichthyidae families (e.g. the genera *Cyclothone* and *Vinciguerria*) were recorded in the Gulf, confirming (with small deviations) previous knowledge on species composition in the area, which was based on ichthyoplankton surveys^26^. The only deviations from previous findings were (i) the presence of *Notoscopelus *sp. which had not been reported in the area, and (ii) the absence of the myctophid *Lobianchia dofleini*, which had been recorded as larvae in the past^26^. The former could be attributed to the timing of the larval study that probably did not coincide with the reproductive period of the species. The species composition of the Gulf of Corinth is discrete compared to other deep seas of the Central Mediterranean^26,27,49^. This, along with possible differences in species’ growth and population structure^50^, highlight the need to study mesopelagic fish at the regional scale.

Still, the presence of > 15 taxa in the area is interesting given that the Gulf of Corinth is geographically isolated from adjacent deep seas essentially since its formation^51^, which may hinder horizontal movements, population exchange and even gene flow^52^. It should be noted that during the last glacial period the sea level was much lower and the deeper part of the Gulf was an isolated lake; even today, its distance to the nearest deep (> 400 m) region of the adjacent Ionian Sea is about 80 nmi. Although the deep-water masses of the gulf have distinct characteristics (lower temperature and salinity) than the open Ionian Sea^28^ they don’t seem capable to constraint species’ presence. On the other hand, there is a limited exchange of water bodies (especially deep ones) between the two areas due to their narrow and shallow connection^28^ (minimum < 50 m). Therefore, as already suggested^26^, this isolation is the most plausible explanation for the absence of deep-living (> 400 m)^25^, non-migratory genera (especially *Cyclothone* sp.) and for the fact that the mesopelagic ichthyofauna of the Gulf of Corinth mostly includes vertically migratory species. Such species are probably more effective in overcoming the shallow-regions obstacle through active horizontal migration during the night^53^ as adults, which may further favour passive transport of epipelagic eggs and larvae due to of shorter distance and transit time. At the species level, the duration of larval stages and the existence of suitable habitats (e.g. temperature and salinity conditions) at time of settlement influence larval survival and population connectivity^54^. This information however is scarce for the majority of species and more work is needed on population connectivity and the underlying factors.

In fact, of all the mesopelagic fish recorded, only three species do not perform extensive DVM. *Argyropelecus hemigymnus* is considered as partial^55^ or non-migrator with a bimodal depth distribution^56^ as also found in the current study. In addition, *D. holti* and *L. crocodilus* were not found in the upper layers during night and don’t seem to perform extensive migrations either. The DSL that *A. hemigymnus* and *D. holti* inhabit was mainly found in the autumn surveys and the species didn’t show sign of relocation closer to the surface at dusk, as confirmed by the day/night acoustic backscatter and also catch data. Interestingly, these two species have been also found together in distinct layers in the Western Mediterranean^15^, albeit at larger depths (around 400m instead of ~ 250 in our study), which could be due to differing optical and other environmental conditions^57^.Regarding *M. muelleri, it* was not targeted at night and no conclusion can be derived, although the species has been demonstrated to perform DVM in the Atlantic^e.g.,^^58^. All the remaining species inhabiting the deeper DSL were found to migrate, with part of their population also found at depth during the night. A reduction of 30% in the DSL’s average NASC was estimated during the night, a portion most likely biased due to lower SNR and detection capability at higher ranges. The most abundant species found in the upper layer during the night were *C. maderensis, H. benoiti* and *B. glaciale,* in line with what has been previously evidenced in the W. Mediterranean^23,25^. Additionally, although large numbers of individuals of *M. punctatum* were observed on the sea surface at night (and caught with a landing net; authors’ pers. observation), they were not effectively sampled, probably due to their very shallow positioning and consequent sampling gear escape. The size-related patterns in bathymetric distribution and DVM were species-specific. For *B. glaciale* and *Notoscopelus *sp. smaller fish were more associated with DVM, in line with results from fjords (*B. glaciale*)^59^ and the west Mediterranean (*Notoscopelus *sp.)^15^, where larger individuals were generally distributed deeper at night. For several mesopelagic species, larger individuals have been shown to adopt a more benthopelagic non-migratory behaviour, which has been associated with seamounts^60^. However, such patterns are not confirmed for all mesopelagic species inhabiting the Gulf of Corinth since for *C. maderensis*, *H. benoiti* and *Lestidiops *sp. it was the larger individuals that were shown to migrate more. In any case, there seems to be high plasticity in DVM patterns which may change seasonally and a more targeted sampling may be needed to elucidate more on species’ DVM behaviour^59^.

Biological sampling in the mesopelagic zone is indeed an issue of ongoing research^18^ and no standard sampling gears have been agreed or even proposed in coordinated surveys^19^. The current sampling offered the opportunity to perform a limited comparison in size-related catch efficiency of three sampling gears, i.e. two pelagic trawls and one frame sampler. As expected because of its smaller mesh size, the Methot frame was more effective in catching smaller individuals, but the larger individuals with higher swimming ability were able to avoid it due to its smaller opening and lower towing speed. The two pelagic trawls caught similar size ranges for most species, but the smaller Sardonet trawl (smaller opening and mesh size) sampled more effectively intermediate sizes of *D. holti* and *H. benoiti* which were present in lower rates in both the larger trawl and the frame. It seems that none of the three gears was adequate to sample all sizes and species, however, the pelagic trawl caught the larger quantities, which is important for the acquisition of biological samples, especially given the substantial time and effort required to sample the deep sea. Therefore, the use of multiple gears seems important for adequate sampling of the mesopelagic community^5^.

Overall, even though biomass estimates have not been produced due to the weaknesses of vessel-mounted echosounders in reliably quantifying backscattering echo from the deep and the high associated uncertainty in existing methodologies^17,19,61^, the extent of the detected DSLs indicate that the Gulf of Corinth sustains high densities of mesopelagic fish. Due to the relatively low abundance of small pelagics in the area^33^, which are concentrated over the -very narrow- continental shelf, it is safe to assume that mesopelagic fish constitute the basic food resource for the dolphins populations that inhabit the Gulf; especially the very abundant (~ 1300 individuals^62^) striped dolphins and the critically endangered common dolphins^32^ that inhabit the deeper parts of the Gulf^30^ and largely overlap with the distribution of the DSLs. Although quantitative information from the area does not exist, both cetacean species have been reported to feed extensively on mesopelagic fish in other regions of the Mediterranean^63,64^. As mesopelagic fish have been found to constitute important part of the diet of commercial species as well^e.g.^^65,66^, ecological interactions related to them should be taken more into account under an Ecosystem Approach to Fisheries. In this direction, due to its bathymetry, isolation, proximity to the coast and the limited adverse weather conditions compared to the open seas, the Gulf of Corinth could serve as an ocean laboratory, similarly to fjords^67^, in the effort to advance knowledge on mesopelagic fish and their ecological role.

Acoustics effectively detected different echo-types, with the echotraces belonging to *M. muelleri* being completely distinct compared to the remaining DSLs, a feature also reported elsewhere^68^. The observed number of distinct DSLs and their characteristics also differed seasonally. This was probably due to seasonal differences in the length composition and the relative abundance of the DSL-forming populations. The spawning period of most mesopelagic species is not well studied; many fishes spawn throughout the year or for an extensive period^3^, nevertheless, the seasonal patterns in population structure detected here, imply seasonality in spawning and recruitment of several species. This seasonality is also supported by the fact that the autumn surveys (November 2018 and October 2019) showed similarities not only in environmental conditions but also in length frequencies of the species and the characteristics of the DSLs, compared to the spring survey which stood out. Seasonal differences in mesopelagic fish composition and behaviour are not uncommon^14,69^, however the mechanisms behind these should be further studied to disentangle the effect of intrinsic (e.g. growth, reproduction, life history stage)^70^ and extrinsic parameters such as food availability, competition, sun inclination, light availability and duration, water stratification^71^.

The current research sheds light on the diversity, population characteristics, spatial distribution and behavior of the mesopelagic fish community with implications on their importance in the environment of the Gulf of Corinth. Despite the new knowledge added, important issues remain to be clarified for future works. The seasonal, vertical and horizontal distribution of each species should be further explored under the prism of different biological characteristics (e.g. maturity and reproduction). Furthermore, questions should be addressed regarding interannual differences in abundance and possible association with environmental parameters. Finer understanding of the depth distribution and DVM per species is also needed with special focus on the DVM patterns of *M. muelleri* which was shown to form distinct schools and shoals, occupying a different habitat than the remaining mesopelagic fishes and is known to demonstrate interesting migration patterns elsewhere^58^.

### Supplementary Information

Supplementary Information.

## Data Availability

Raw biological sampling catch data are available in the submitted tables (in the main text and the supplementary material). Due to their size, individual fish measurements and raw acoustic data used in the analysis are available from the corresponding author on reasonable request.
