# Supplementary material for: Seasonal patterns in the mesopelagic fish community and associated deep scattering layers of an enclosed deep basin
Source: Sci Rep. 2023 Oct 19;13:17890. doi: 10.1038/s41598-023-44765-5 (PMC10587179; doi:10.1038/s41598-023-44765-5)
Supplement: Supplementary file 1 — Supplementary Information. [file 41598_2023_44765_MOESM1_ESM.docx]

**Seasonal patterns in the mesopelagic fish community and associated deep scattering layers of an enclosed deep basin.**

**Authors:** Z. Kapelonis, A. Siapatis, A. Machias, S. Somarakis, K. Markakis, M. Giannoulaki, N. Badouvas, K. Tsagarakis

**Supplementary Material**


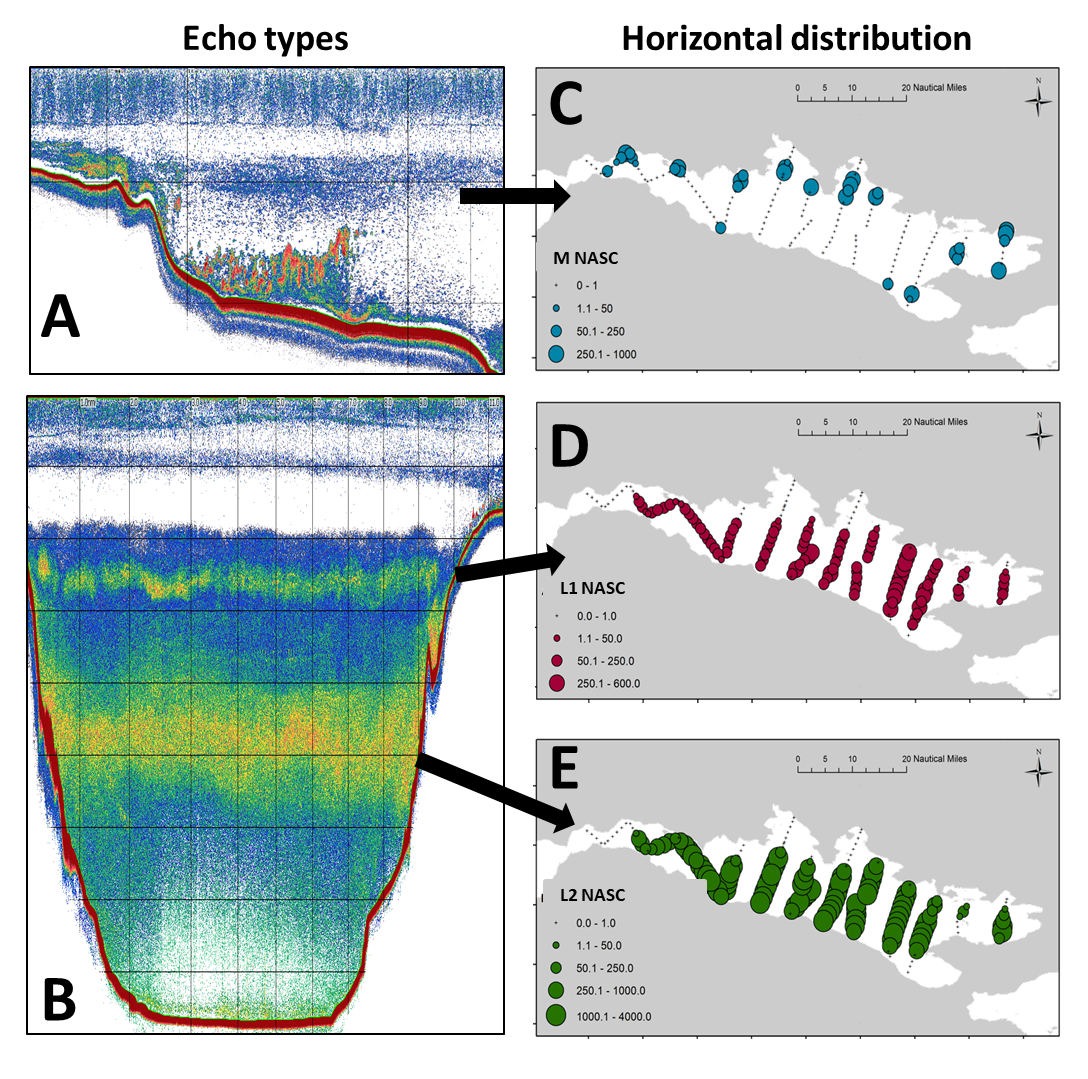


**Supplementary Figure S1.** Horizontal distribution of the main echo-types in October 2018. (A) Echo-type M, i.e. schools and shoals formed by *Maurolicus muelleri*, (B) L1 and L2 DSLs formed by *Argyropelecus hemigymnus* and a mixture of species respectively, (C-E) spatial distribution of Μ, L1 and L2 echo-types. Bubble size represents total NASC per EDSU.

**Supplementary Table S1.** Methot frame catch composition (number of individuals) and towing characteristics. Juvenile individuals are indicated with “(juv.)” while the remaining specimens are larvae. Juveniles were considered the individuals with all photophores developed.

|  | **Cruise** | **Nov 2018** |  |  |  | **Apr 2019** | **Oct 2019** |
| --- | --- | --- | --- | --- | --- | --- | --- |
|  | **D/N** | **D** |  | **N** |  | **D** | **D** |
|  | **Depth of station (m)** | **846** | **858** | **832** | **861** | **530** | **834** |
|  | **Haul depth (m)** | **227** | **406** | **16** | **509** | **86** | **264** |
|  | **Duration Of haul (min)** | **66** | **62** | **51** | **51** | **29** | **48** |
|  | **Volume sampled (m^3^)** | **8709.03** | **8181.21** | **7438.095** | **6729.705** | **4229.505** | **6333.84** |
| **Family** | **Species \| Station** | **Methot 1** | **Methot 2** | **Methot 3** | **Methot 4** | **Methot 1** | **Methot1** |
| **Argentinidae** | *Glossanodon leiglossus* |  |  |  |  | 1 |  |
| **Bothidae** | *Arnoglossus sp.* |  |  | 1 | 1 | 1 | 2 |
| **Capridae** | *Capros aper* | 1 |  |  | 1 |  |  |
| **Carangidae** | *Trachurus trachurus* |  |  |  |  | 2 |  |
| **Centrolophidae** | *Centrolophus niger* |  |  | 2 | 1 |  |  |
| **Cepolidae** | *Cepola macrophthalma* |  |  | 3 | 2 |  |  |
| **Clupeidae** | *Sardina pilchardus* |  |  |  |  | 34 |  |
| **Congridae** | *Ariosoma balearicum* |  |  |  | 1 |  |  |
|  | *Conger conger* | 3 |  |  | 3 |  |  |
|  | *Gnathophis mystax* |  |  |  | 4 |  | 2 |
| **Cynoglossidae** | *Symphurus nigrescens* | 3 |  |  | 1 |  | 4 |
| **Labridae** | *Coris julis* |  |  |  | 1 |  | 1 |
| **Myctophidae** | *Benthosema glaciale* |  |  |  |  | 60 |  |
|  | ***Benthosema glaciale (juv.)*** |  | 74 | 24 | 30 |  |  |
|  | *Ceratoscopelus maderensis* | 4 | 6 | 94 | 215 |  | 28 |
|  | ***Ceratoscopelus maderensis (juv.)*** |  | 148 | 64 | 23 |  |  |
|  | *Diaphus holti* | 8 | 2 | 5 | 15 |  | 8 |
|  | ***Diaphus holti (juv.)*** |  | 26 | 2 | 3 |  |  |
|  | *Hygophum benoiti* | 18 | 7 | 110 | 117 | 57 | 19 |
|  | ***Hygophum benoiti (juv.)*** |  | 3 | 4 | 87 |  |  |
|  | *Lampanyctus crocodilus* |  |  |  | 5 | 10 | 1 |
|  | ***Lampanyctus crocodilus (juv.)*** |  | 8 |  | 1 |  |  |
|  | *Myctophum punctatum* |  |  |  |  | 97 |  |
|  | ***Myctophum punctatum (juv.)*** |  | 1 |  |  |  |  |
| **Nettastomatidae** | *Faciolella oxyrhyncha* |  |  | 1 |  |  |  |
| **Paralepididae** | *Arctozenus risso* |  | 2 |  | 2 | 21 | 1 |
|  | *Lestidiops jayakari* | 9 |  |  | 5 | 17 | 1 |
| **Pomacentridae** | *Chromis chromis* |  |  |  |  |  | 2 |
| **Scorpaenidae** | *Scorpaenidae* |  |  |  |  |  | 2 |
| **Serranidae** | *Anthias anthias* |  | 2 |  | 1 |  | 1 |
| **Soleidae** | *Microchirus variegatus* |  |  |  | 1 |  |  |
| **Sparidae** | *Boops boops* |  |  |  |  | 15 |  |
|  | *Pagellus acarne* | 4 |  | 2 | 2 |  |  |
| **Sphyraenidae** | *Sphyraena sphyraena* |  |  |  |  | 1 |  |
| **Sternoptychidae** | *Argyropelecus hemigymnus* | 14 | 1 | 1 | 56 | 6 | 12 |
|  | ***Argyropelecus hemigymnus (juv.)*** | 977 | 64 | 8 | 2 |  | 479 |
|  | *Maurolicus muelleri* | 1 |  | 2 | 3 | 514 |  |
| **Stomiidae** | *Stomias boa* | 1 |  |  | 2 | 10 | 1 |
| **Trichiuridae** | *Lepidopus caudatus* | 1 |  | 2 | 2 |  | 1 |
| **Total** |  | **1044** | **344** | **325** | **587** | **846** | **565** |


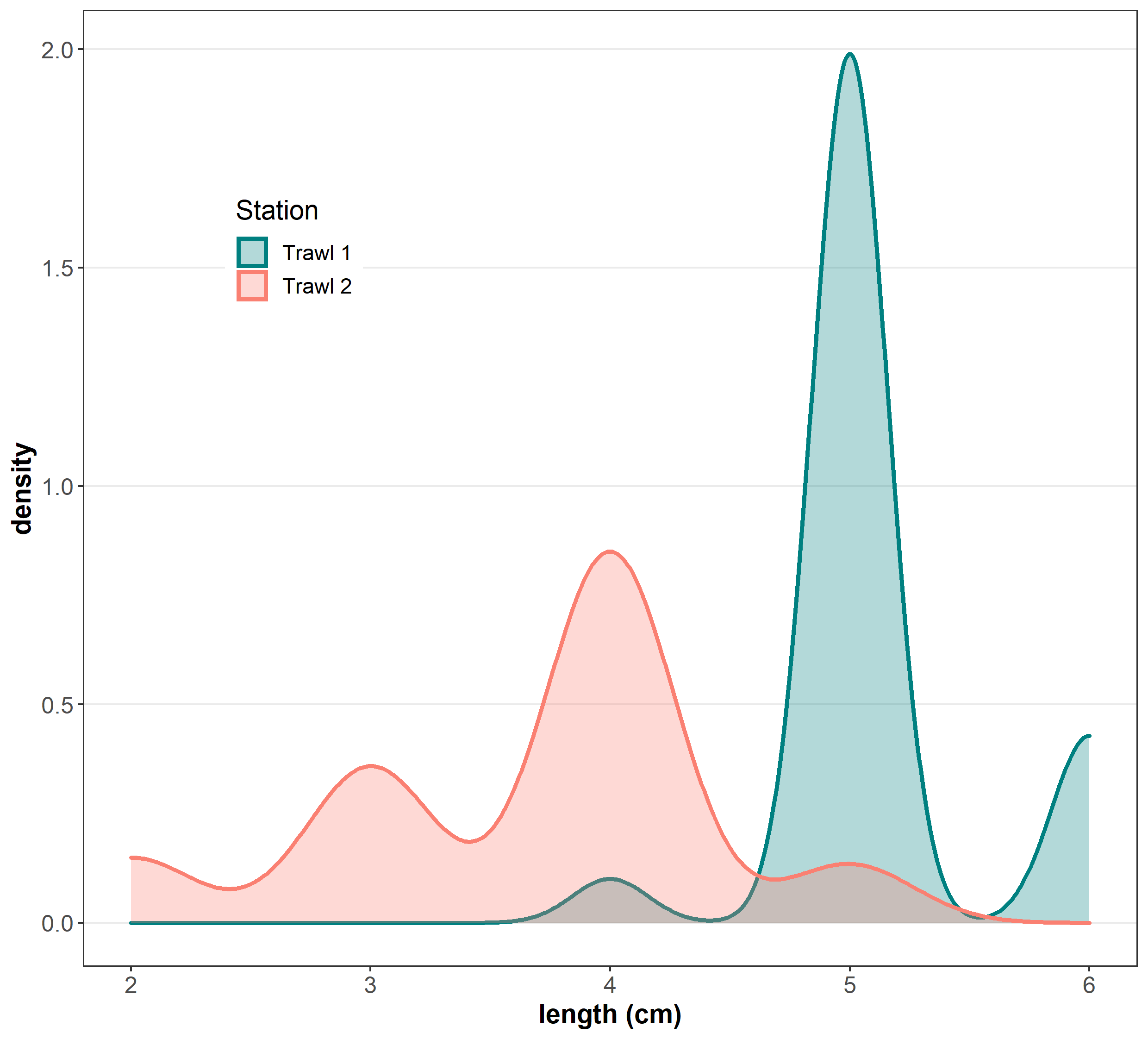


**Supplementary Figure S2.** Density plots of lengths of M. muelleri in April 2019 in two different bathymetric layers, at ~175 m (Trawl 1) and ~130 m depth (Trawl 2).
